# Discovery of Novel Anti‐Resistance AR Antagonists Guided by Funnel Metadynamics Simulation

**DOI:** 10.1002/advs.202309261

**Published:** 2024-03-13

**Authors:** Haiyi Chen, Yuxin Zhou, Xinyue Wang, Xin Chai, Zhe Wang, Ercheng Wang, Lei Xu, Tingjun Hou, Dan Li, Mojie Duan

**Affiliations:** ^1^ College of Pharmaceutical Sciences Zhejiang University Hangzhou Zhejiang 310058 China; ^2^ National Centre for Magnetic Resonance in Wuhan State Key Laboratory of Magnetic Resonance and Atomic and Molecular Physics Innovation Academy for Precision Measurement Science and Technology Chinese Academy of Sciences Wuhan Hubei 430071 China; ^3^ Liangzhu Laboratory Zhejiang University Medical Center Hangzhou Zhejiang 311121 China; ^4^ Zhejiang Laboratory Hangzhou Zhejiang 311100 China; ^5^ Institute of Bioinformatics and Medical Engineering School of Electrical and Information Engineering Jiangsu University of Technology Changzhou 213001 China; ^6^ NMR and Molecular Sciences, School of Chemistry and Chemical Engineering, The State Key Laboratory of Refractories and Metallurgy Wuhan University of Science and Technology Wuhan 430081 China

**Keywords:** antagonist, Funnel metadynamics, molecular docking, prostate cancer

## Abstract

Androgen receptor (AR) antagonists are widely used for the treatment of prostate cancer (PCa), but their therapeutic efficacy is usually compromised by the rapid emergence of drug resistance. However, the lack of the detailed interaction between AR and its antagonists poses a major obstacle to the design of novel AR antagonists. Here, funnel metadynamics is employed to elucidate the inherent regulation mechanisms of three AR antagonists (hydroxyflutamide, enzalutamide, and darolutamide) on AR. For the first time it is observed that the binding of antagonists significantly disturbed the C‐terminus of AR helix‐11, thereby disrupting the specific internal hydrophobic contacts of AR‐LBD and correspondingly the communication between AR ligand binding pocket (AR‐LBP), activation function 2 (AF2), and binding function 3 (BF3). The subsequent bioassays verified the necessity of the hydrophobic contacts for AR function. Furthermore, it is found that darolutamide, a newly approved AR antagonist capable of fighting almost all reported drug resistant AR mutants, can induce antagonistic binding structure. Subsequently, docking‐based virtual screening toward the dominant binding conformation of AR for darolutamide is conducted, and three novel AR antagonists with favorable binding affinity and strong capability to combat drug resistance are identified by in vitro bioassays. This work provides a novel rational strategy for the development of anti‐resistant AR antagonists.

## Graphical abstract

By utilizing funnel metadynamics (FM), the underlying mechanism of AR antagonism is elucidated. The FM‐derived representative binding conformation of ODM201 is considered insensitive toward common drug‐resistant AR mutants. Leveraging this information, three novel antagonists with anti‐resistant activity are discovered through docking‐based virtual screening and bioassay methods.

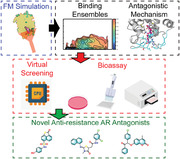

## 1 Introduction

**Figure 1:**
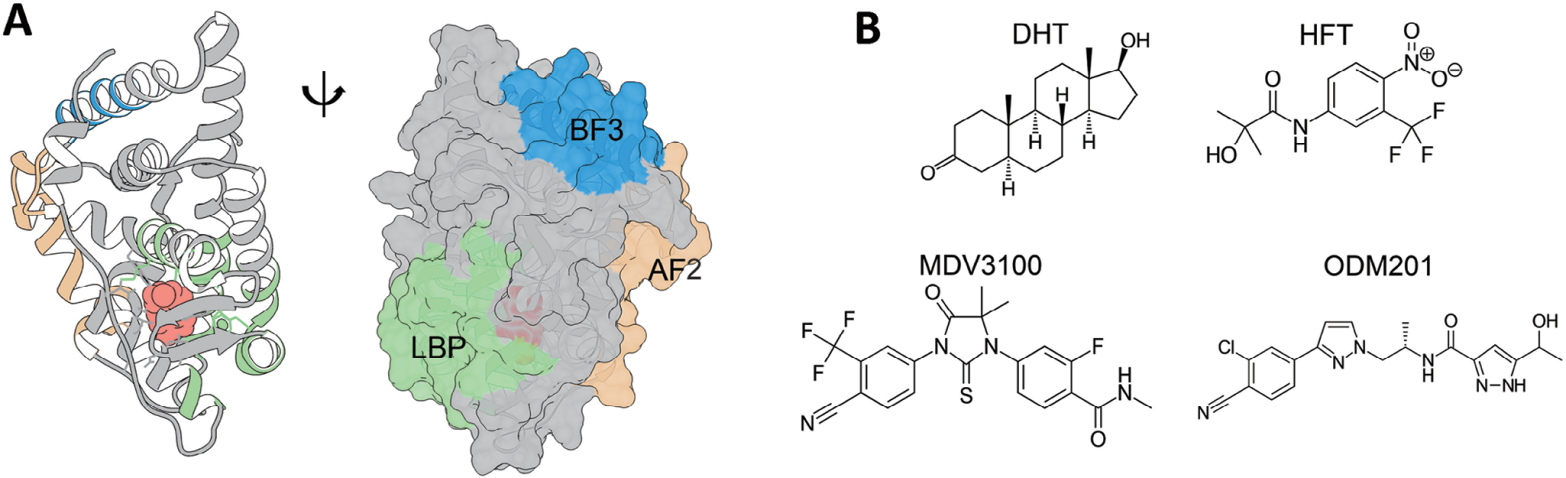
The activated structure of the AR‐LBD (PDB: 1T5Z) and ligands used in this study. A) The AR‐LBD structure bound with the agonistic ligand dihydrotestosterone (DHT); B) The chemical structures of the ligands used in this study. DHT: dihydrotestosterone; HFT: hydroxyflutamide; MDV3100: enzalutamide; ODM201: darolutamide.

Prostate cancer (PCa) is one of the most diagnosed cancers in men worldwide. Aberrant activation of androgen receptor (AR) is closely related to the occurrence and progress of PCa.^[^ ^1^ ^]^ AR is a member of the nuclear receptor superfamily and consists of four distinct functional domains: the N‐terminal domain (NTD), the DNA‐binding domain (DBD), the hinge region, and the ligand binding domain (LBD).^[^ ^2^ ^]^ The receptor is normally located in the cytoplasm and remains inactive by binding with heat shock proteins (HSPs) and cytoskeletal proteins. Previous studies show that upon the binding of endogenous androgens such as dihydrotestosterone (DHT) to the ligand binding pocket (LBP) at the AR‐LBD, conformational changes occur in helix‐3, 4, and 12, thereby stabilizing the AF2 binding surface (**Figure** **1**A).^[^ ^3^ ^]^ Simultaneously, molecular chaperones (such as HSPs) dissociate from AR. Guided by specific proteins, AR then translocates into the nucleus^[^ ^4^ ^]^ and binds to androgen response elements (AREs) as homodimer on DNA.^[^ ^5^ ^]^ Here the AF2 site recruits coregulators and other proteins to form a transcriptional regulatory complex.^[^ ^2^, ^3^, ^6^ ^]^ Current strategies for the treatment of PCa typically involve inhibiting androgen synthesis (chemical castration) and blocking AR transcriptional activity.^[^ ^7^ ^]^ The latter normally refers to antagonists which competitively bind to the AR‐LBP. There are two generations of AR antagonists assigned to clinical therapy of PCa, with the second generation showing better effect in blocking AR transactivation. However, drug resistant mutations such as H874Y, F876L, and T877A have been reported.^[^ ^8^ ^]^ The mutations lead to the activation of AR by the first generation of AR antagonists such as flutamide or the second generation of AR antagonists such as enzalutamide (MDV3100),^[^ ^8^ ^]^ which greatly hinders the clinical application of AR antagonists. Although newly approved darolutamide (ODM201) shows antagonistic activity to the above mutants, it still faces a large risk of drug resistance in the forthcoming days. As the mechanism by which specific mutations induce the conversion of antagonists into agonists has not been well elucidated, some researchers have shifted their focus toward other potential sites of AR, including NTD,^[^ ^9^ ^]^ DBD,^[^ ^10^ ^]^ AF2,^[^ ^11^ ^]^ BF3,^[^ ^12^ ^]^ and the homo‐dimeric interface of the AR‐LBD.^[^ ^13^ ^]^ However, up to today, all approved AR antagonists do target the AR‐LBP rather than the potential sites above, which strongly suggests that AR‐LBP is still the only time‐honored targeting site of AR antagonists. Therefore, the development of structurally diverse AR antagonists targeting the AR‐LBP is requisite especially for fighting against drug resistance.

In recent years, computer aided drug design (CADD) has been successfully applied to identify new‐scaffold AR antagonists even when resolved structure of wild‐type AR bound with antagonists which maintains antagonistic conformation of AR is still lacking.^[^ ^14^ ^]^ Nevertheless, whether the antagonists are active to AR mutants could not be well predicted theoretically due to deficient knowledge of the regulation mechanism of AR by antagonists at atomistic level. In order to fill the gap, computational chemists have primarily focused on elucidating two key questions: 1) how antagonists regulate the conformation of the AR‐LBD to render it inactive, and 2) how specific residue mutations result in the conversion of certain antagonists into agonists. For the first question, in 2016, we conducted extensive unbiased molecular dynamics (MD) simulations and bias‐exchange metadynamics (BE‐MetaD) to investigate the regulatory effects of antagonists such as HFT and bicalutamide on the AR‐LBD,^[^ ^3b^ ^]^ and the results revealed that antagonists partially disrupt the secondary structure of helix‐12 compared to agonists and the residues W741, H874, and I899 are involved in the regulation of the helix‐12 conformation by antagonists. In 2018, Liu et al. further demonstrated that the ligand binding to AR‐LBP not only regulates the conformation of the AF2 region but also affects another site called the binding function 3 (BF3),^[^ ^15^ ^]^ which has been shown to be related to AR activity.^[^ ^12^, ^16^ ^]^ By utilizing unbiased MD simulations and MM/GBSA calculations, Ye et al. found that the binding of agonists stabilizes the AF2 conformation, making it more likely to incorporate coactivators,^[^ ^17^ ^]^ and this, in turn, enhances the interaction between agonists and the AR‐LBD. However, this phenomenon was not observed in the complex of the AR‐LBD with HFT. These studies pointed out the differences in the AF2 region between agonist and antagonist binding, but the limited sampling of the AR‐LBD could not provide a clear explanation for this difference.

Similarly, the theoretical works on the second question appear to be highly circumscribed, as these studies are case‐specific and the sampling of the binding conformations is usually insufficient. Through replica exchange molecular dynamics (REMD) simulations, Zhou et al. revealed that the T877A mutant of the AR‐LBD adopts an agonistic conformation of helix‐12 upon the binding with HFT,^[^ ^18^ ^]^ consistent with our observation.^[^ ^3b^ ^]^ In 2017, Liu et al. constructed the structures of HFT in complex with the wild‐type AR and several major resistance‐related AR mutants.^[^ ^19^ ^]^ Using conventional MD simulations and MM/GBSA calculations, they found that the hydrogen bonding interaction between HFT and N705 was significantly enhanced in the AR mutants with T877A, W741C/T877A, and F876L/T877A. In another study published by Liu et al., similar phenomenon was observed with MDV3100,^[^ ^20^ ^]^ where specific point mutations led to significantly enhanced hydrogen bonding interactions between MDV3100 and Q711 or R752. By utilizing molecular docking and long‐duration MD simulations, Azhagiya et al. found that bicalutamide and HFT, which can be converted to agonists under specific circumstances, and AR agonists such as DHT and testosterone may interact with N705 and R752 through hydrogen bonding.^[^ ^21^ ^]^ It should be noted that the conclusions drawn from the above studies are greatly influenced by the initial binding conformations of ligands and the sampling adopted might be unable to cover the binding conformational space for various ligands.

In this study, we utilized the well‐developed sampling method funnel metadynamics (FM)^[^ ^22^ ^]^ to sample the binding of the ligands in a totally different way from previous studies, enabling the ligand to bind to the AR‐LBP and then dissociate repeatedly to generate a complete binding ensemble. Since being reported in 2013,^[^ ^22^ ^]^ the FM method has been applied in accurately calculating the binding free energies for different ligands,^[^ ^23^ ^]^ but its ability to explore the ligand‐binding conformational space has not yet been fully explored. The binding structures of three structurally diverse AR antagonists, HFT (the active metabolite of the first generation AR antagonist flutamide), MDV3100 (the second generation AR antagonist), and ODM201 (the newly approved AR antagonist) were comprehensively analyzed with the endogenous AR agonist DHT as a control (Figure 1B). The selection of these three antagonists as subjects is based on the following reasons: i) the structural types of these three antagonists are quite different from each other, and ii) while HFT and MDV3100 are susceptible to typical resistance mutations, ODM maintains antagonistic activity against these mutations, despite the unclear reasoning behind this distinction. The question of how antagonists regulate the conformation of the AR‐LBD to render it inactive was satisfactorily answered by our study. Based on the free energy landscapes calculated by FM, we found that the internal hydrophobic contacts of specific residues, as well as the cooperation between three known functional sites AR‐LBP, AF2, and BF3 (Figure 1A) were strengthened upon the binding of DHT but disrupted upon the binding of antagonists. Further bioassays verified the necessity of these hydrophobic contacts to AR activity. In addition, non‐antagonistic binding conformations were identified in a considerable portion for HFT and MDV3100, while only antagonistic binding conformations were collected for ODM201, indicating the correlation between anti‐resistance activity and the binding conformational type. Then based on the representative binding conformation of the AR‐LBP with ODM201, we performed virtual screening (VS) and selected 80 compounds for experimental testing. Three novel AR antagonists (i.e., HY25, HY27, and HY60) with good capability to combat major drug‐resistant mutations were identified. The specific facts may help to answer the question of how mutations in specific residues result in the conversion of certain antagonists into agonists in the future. In summary, our work provides an important reference for the development of novel antagonists targeting the LBP of nuclear receptors.

## 2 Results and Discussion

### 2.1 Binding Free Energies of AR‐LBP Ligands Calculated by FM Sampling are in Agreement with Measurement

Previous computational studies on AR/ligand interactions primarily relied on available crystal structures of complexes or docking models. In this study, we employed the FM method, which periodically incorporates a Gaussian bias potential within a funnel‐shaped restraint to enable the ligand to explore the binding conformations globally along the selected reaction coordinates. Once convergence is reached, the bias potentials are subtracted from the free energy landscape to accurately recover the binding free energy of the ligand. This method requires the pre‐knowledge of the ligand's entry or exit pathways to set up the cylindrical region for the funnel restraint. Previous studies have indicated that the ligand entry pathway into the nuclear receptor involves a channel between helix‐3, helix‐7, and helix‐11.^[^ ^24^ ^]^ Considering the possibility of alternative pathways for ligand dissociation from the nuclear receptor,^[^ ^25^ ^]^ we first used the random accelerated MD (RAMD)^[^ ^26^ ^]^ method to explore the dissociation pathways of the four ligands from the AR‐LBP. Each system was run with 40 parallel replicas, and the results are shown in Table S1 (Supporting Information). For all four ligands, the conservative channel formed by helix‐3, helix‐7, and helix‐11 was identified as the primary dissociation pathway, since DHT, HFT, MDV3100, and ODM201 dissociated from this channel 28, 26, 40, and 29 times, respectively. Based on the RAMD results, we set up the funnel restraint of FM along this channel, as shown in Figure S1 (Supporting Information). Following the computational method proposed by Limongelli et al.,^[^ ^22^ ^]^ we calculated the binding free energies (Δ*G*) and binding affinities (*K_d_*) of the four ligands with the AR‐LBP (**Table** **1**). The *K_d_* value of DHT (3.81 ± 0.19 nm) matches the experimental value (3.4 nm) very well. There are no experimental reports on the *K_d_* values for the other three antagonists, and the theoretical *K_d_* values predicted by our study are 10.49 ± 0.83, 7.64 ± 1.55, and 5.23 ± 0.34 nm for HFT, MDV3100 and ODM201, respectively, which are in the same order of magnitude as the corresponding experimental *K_i_* values (43, 28, and 11 nm for HFT, MDV3100, and ODM201, respectively) and exhibit a clear positive correlation with *K_i_*. These results indicate that our FM calculation results based on the H3‐H7‐H11 pathway are reasonable when compared with the real cellular circumstance.

**Table 1:** The binding affinity and binding free energies of the ligands to the AR‐LBD.

Ligand	*K*_d_ ^a)^	Δ*G* ^b^ ^)^	*K*_d_ (exp)^a^ ^)^	*K*_i_ (exp)^c)^	Δ*G* (exp)^b)^
DHT	3.81 ± 0.19	−12.60 ± 0.03	3.4	n.a.^d)^	−12.2
HFT	10.49 ± 0.83	−12.01 ± 0.05	n.a.	43	n.a.
MDV3100	7.64 ± 1.55	−12.20 ± 0.11	n.a.	28.2	n.a.
ODM201	5.23 ± 0.34	−12.43 ± 0.02	n.a.	11	n.a.

^a)^ *K_d_* and *K*_i_ in the unit of nm. *K*_x_(exp) is corresponding to the experimental measurements;

^b)^ Δ*G* in the unit of kcal mol^−1^;

^c)^ *K_i_* of HFT measured the Inhibition of [3H]‐DHT binding to androgen receptor of MDA‐453 cells,^[^ ^27^ ^]^ and MDV3100 measured the antagonist activity at androgen receptor,^[^ ^28^ ^]^ ODM‐201 measured in competition with [3H]‐mibolerone using wild‐type AR isolated from rat ventral prostates;^[^ ^29^ ^]^

^d)^ Not available.

### 2.2 Channel H3‐H7‐H11 is Significantly Regulated by the Ligands into Distinct Modes

**Figure 2:**
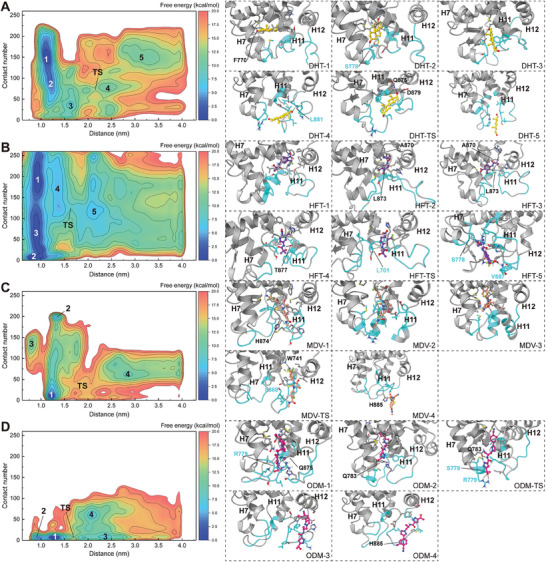
The free energy surface related to the binding/unbinding processes of DHT/HFT/MDV3100/ODM201 to the AR‐LBD A–D respectively). The x‐axis corresponding to the center‐of‐mass (COM) distances between protein and ligand. The y‐axis values represent the number of the native contact atom pairs of hydrophobic residues in the binding portal region of the AR‐LBD. The free energy minima are labeled and the representative structures of free energy minima are given. The transition states are estimated based on the saddle points on the free energy surfaces. The AR‐LBDs are present in cartoon and the ligands are shown in ball‐and‐stick. The CV2‐relevant residues are colored cyan.

Up to now, no report has been published regarding the complex structure of the wild‐type AR‐LBD bound with any antagonist, making it impossible to ascertain how antagonists regulate the conformation of the AR‐LBD. In this study, we attempted to address the issue raised in the introduction section, that is, how antagonists regulate the conformation of the AR‐LBD to render it inactive. In the FM protocol, two reaction coordinate or collective variable (CVs) were defined to describe the binding of the ligands to the AR‐LBD. The first CV1 represented the distance between the center of mass of ligand and that of the AR‐LBD. The second CV2 (calculated based on the atom‐pairs that were in native contact within 4.5 Å in the crystal structure of AR‐DHT, and denoted as contact number) represented the residue contact values within the channel formed by helix‐3, helix‐7, and helix‐11. Higher value of contact number indicates a more closed and compact conformation of the channel. Based on these two CVs, the free energy landscape of each ligand‐AR‐LBD complex was plotted, as shown in **Figure** **2**. Distinct minima corresponding to the bound, transition, and unbound states can be distinguished on each landscape. The conformations within each minimum represented an ensemble of the states associated with specific binding modes. For DHT, the conformations within Minimum 1 were defined as its bound state conformations (Figure 2A). For HFT, the conformations within **Minima 1, 2, and 3** were defined as its bound state conformations (Figure 2B). For MDV3100, the conformations within **Minima 1** and **2** were defined as its bound state conformations (Figure 2C). For ODM201, the conformations within **Minimum 1** were defined as its bound state conformations (Figure 2D).

First, we noticed that the contact value distributions of the binding states of the four ligands are significantly different. The bound state of DHT was associated with a relatively larger contact number, ≈150 **(Minimum1**, Figure 2A). As shown in Figure 2B, the contact number values corresponding to the three bound states of HFT were ≈170–220 **(Minimum 1)**, 50–90 **(Minimum 3)**, and below 10 **(Minimum 2)**, respectively. For MDV301 (Figure 2C), the two bound states had the contact number values of ≈200 **(Minimum 2)** and below 20 (**Minimum 1**), respectively. The bound state conformation of ODM201 was only associated with a contact number below 15 **(Minimum 1**, as shown in Figure 2D). This indicates that the binding of the four ligands exert distinct effects on the conformation of the AR‐LBD.

**Figure 3:**
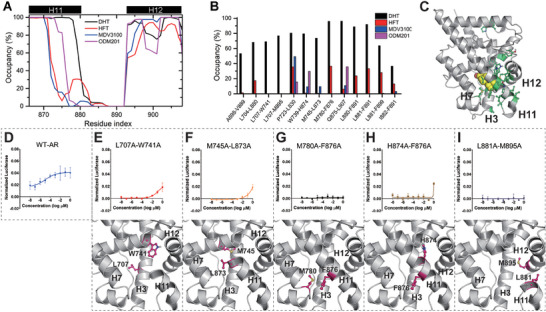
The regulation of ligands to the AR‐LBD in the ligand‐bound state. A) The helicity of residues in the helix‐11 and helix‐12 regions. B) The contact occupancy of hydrophobic residues in the AR‐LBD of ligand‐bound ensembles. C) The distribution of the residue pairs listed in Figure 3B. The ligand DHT is drawn as the yellow spheres, while the residue pairs are drawn as the green ball‐stick model. D) The transcriptional activities of the WT‐AR under different concentration of DHT. E–I) The transcriptional activities of the five mutants under different concentration of DHT. Mutated residues are highlighted in pink cartoon diagrams.

Then we tried to verify the specific differences in the local conformation of the AR‐LBD upon the binding of different ligands. The bound state conformation ensembles of each system were collected for the calculation of the folding degree of helix‐12 and the adjacent C‐terminus of helix‐11, as shown in **Figure** **3**A. Surprisingly, the differences in the folding degree of helix‐12 between the four systems were not the most pronounced, though the helix has drawn much attention in previous studies.^[^ ^3^, ^17^ ^]^ Instead, a striking difference was observed in the C‐terminal region (872–879) of helix‐11 between DHT and the three antagonists. In the case of the DHT binding, this segment maintained a folded conformation almost 100% of the time, while in the presence of the three antagonists, it displayed a higher propensity for unfolding (with less than 35% of folded conformation). This observation was consistent with the dominant binding conformations listed in the cartoon diagrams of the ligand‐receptor complexes (Figure 2), as the C‐terminus of helix‐11 appears to be well folded upon the binding of DHT, while in the ensembles of HFT‐2, HFT‐3, MDV‐1, MDV‐2, and ODM‐1, the C‐terminus of helix‐11 is visibly unfolded.

Furthermore, in order to investigate the relationship between the protein conformational changes and the contact changes among residues before and after ligand binding, the contact frequencies of residue pairs within the AR‐LBD (contact distance threshold of 4.5 Å, totally 250 × 250 pairs) were then examined, and the residue pairs with the remarkable contact frequency difference between the bound state conformation ensembles of the four systems were listed. Interestingly, the result showed that most of these residue pairs are hydrophobic pairs, and a significant proportion of these residues is involved in the binding and dissociation channel H3‐H7‐H11 (Figure 3B,C). When DHT bound to AR‐LBP, the contact frequencies of these hydrophobic pairs were notably higher than those when the three antagonists bound, with almost all these frequencies exceeding 60%, as depicted in Figure 3B (black bars). Specifically, the contact frequencies of M780‐F876, L880‐F891, and L881‐F891 exceeded 80%. By contrast, when the three antagonists bound, the above interactions were substantially weakened or even absent, as shown in Figure 3B. This difference implies that these hydrophobic interactions may play an important role in regulating AR activity.

### 2.3 Internal Hydrophobic Contacts and Site Cooperation are Crucial for AR Activity

To validate the FM results above, we designed several AR‐LBD mutants with double mutations to partially disrupt the aforementioned hydrophobic interactions, including M780A/F876A, W739A/L907A, F876/L907A, L881A/M895A, L707A/W741A, M745A/L873A, H874A/F876A, and M895A/F899A. These mutations were manually introduced into the complex of the AR‐LBD and DHT (PDB ID: 1T5Z). Conventional MD simulations of 1 µs were performed on the wild‐type AR‐LBD and the mutants in complex with DHT. During the final 200 ns of the simulations, the RMSD values of the heavy atoms of the AF2 region (compared with the crystal structure) and the ΔRMSF values of the AF2 region (defined in Materials and Methods) were calculated and compared. The results indicate that compared to the wild‐type AR‐LDB, the AF2 regions of the M780A/F876A, L881A/M895A, L707A/W741A, M745A/L873A, and H874A/F876A mutated AR‐LBD exhibited more significant deviations (RMSD > 2.50 Å), and their ΔRMSF values (0.07–0.16 Å) suggested that the AF2 conformations of these five mutants shifted and fluctuated more than that of the wild‐type AR‐LDB, as shown in Table S2 (Supporting Information). Combined with the conclusion of previous studies^[^ ^3^, ^21^ ^]^ that the weakening of the AF2 stability was directly correlated with the decreasing of AR activity, our conventional MD results suggest that the above mutations may disrupt AR activity through affecting the conformation of the AF2 region. To further verify the hypothesis, we expressed the five mutants in AR‐negative cells and tested their transcriptional activity in the presence of DHT. As depicted in Figure 3D, the wild‐type AR displayed a clear dose‐dependent pattern in response to the DHT regulation. By contrast, among the five double mutants, the L707A/W741A, M745A/L873A, and H874A/F876A mutants exhibited transcriptional activity only under high DHT concentrations, while the M780A/F876A and L881A/M895A mutants completely lost their transcriptional activity (Figure 3E–I). Taken together, the FM, MD and bioassay results suggest that the specific internal hydrophobic interactions were closely related to AR transcriptional activity, and disrupting these hydrophobic interactions may remarkably weaken AR activity.

**Figure 4:**
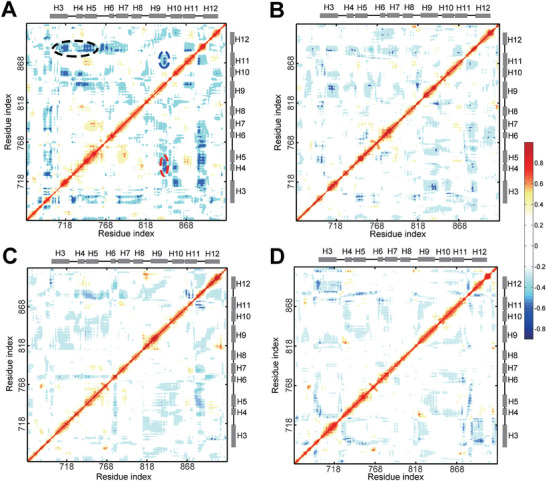
The dynamic cross correlation (DCC) map of the bound structure ensembles of the AR binding with different ligands. A) DHT‐bound AR; B) HFT‐bound AR; C) MDV3100‐bound AR; D) ODM201‐bound AR. Only the grids with dynamic cross correlation larger than 0.4 or less than −0.4 were colored in the figure. The correlation motion with the positive DCC values are colored in red, and the anti‐correlation motions between the residue pairs are colored in blue. The corresponding helices are given aside of the axis, the helices are displayed by the gray bars and the loops connect them are displayed in the black lines.

Notably, the residues above, including L873, H874, F876, L880, L881, I882, and V889, were located at the C‐terminus of helix‐11. F891 was situated at the junction between helix‐11 and helix‐12, while M895 and I899 were positioned on helix‐12. On the other hand, as shown in Figure 1A, the LBP (including the C‐terminus of helix‐11), AF2, and BF3 sites are not spatially adjacent, but all relevant to the activity of AR. We attempted to link the above facts through the correlational analysis of the FM data, so that figure out whether there was an inner cooperation between the LBP, AF2 and BF3. In **Figure** **4**, we present the dynamic cross‐correlation (DCC) map of the AR‐LBD upon the binding of the four ligands. The DCC values reflect the synergy motions between residues, even for the distant residues. The DCC values range from −1 to 1, and the negative values mean the two residues moving in the opposite direction (anti‐correlated motions) and positive values indicate the two residues move in the same direction (correlated motions). The values close to 0 indicate no correlation between two residues. In 2019, Jin et al. utilized DCC analysis to explore the internal cooperation of the AR‐LBP and AF2,^[^ ^17^ ^]^ but the observation based on conventional MD simulations were not quite comprehensive. Herein, our DCC analysis was carried out based on the FM sampling, and the obtained result seemed fascinating, as much stronger anti‐correlated motions were observed in the DHT‐bound AR‐LBD (Figure 4A). Specifically, between residues 708–728 and residues 878–893 (labeled by black dashed circle), a strong anti‐correlation (< −0.6) was present. The residues 708–728 correspond to the C‐terminus of helix‐3, helix‐4, and H3‐H4 loop, which is the AF2 site. The residues 878–893 correspond to the C‐terminus of helix‐11 and H11‐12 loop. That is to say, upon the binding of DHT, there is a non‐negligible cooperation between the AF2 site and the LBP region. Additionally, communication involves the BF3 site (residues 836–840 on helix‐9) and the AF2 site (residues 725–743) is easily observed, as marked by the red dashed circle (Figure 4A). The third correlated pair is the BF3 site (residues 837–840) and helix‐11 (residues 865–870), as marked by the blue dashed circle in Figure 4A. Compared to the binding of DHT, almost all the remote correlations mentioned above were abolished when the antagonists bound to the AR‐LBP (Figure 4B–D). The co‐activator binds to the AF2 region of AR‐LBD via its conserved LXXLL motif, in which M734 on H4 mediates hydrophobic interaction with the coactivator, while K720 on H3 and E897 on H12 anchor the coactivator through polar interactions.^[^ ^30^ ^]^ This stable binding mode is essential for AR activity. Our DCC results revealed that M734 had strong negative correlation motions with BF3 residues (DCC value < −0.4) and K720 had strong negative correlation motions with V887, S888, and V889 of the C‐terminus of H11 (DCC value < −0.5) upon the binding of DHT. This suggests that DHT does not solely regulate AF2 conformation through local direct interactions but also influences the spatial positioning of key AF2 residues by modulating the long‐range residue coupling within AR‐LBD.

Taking all these results into consideration, the DHT binding not only strengthened the contacts between inner hydrophobic residues but also enhanced the inner coupling between the three functional sites, while the binding of antagonists showed the opposite effects. So far, our results are sufficient to answer the question of how antagonists regulate the conformation of the AR‐LBD to render it inactive.

### 2.4 Induced‐Fit Effects of Antagonists Decide their Antagonistic Activity

**Figure 5:**
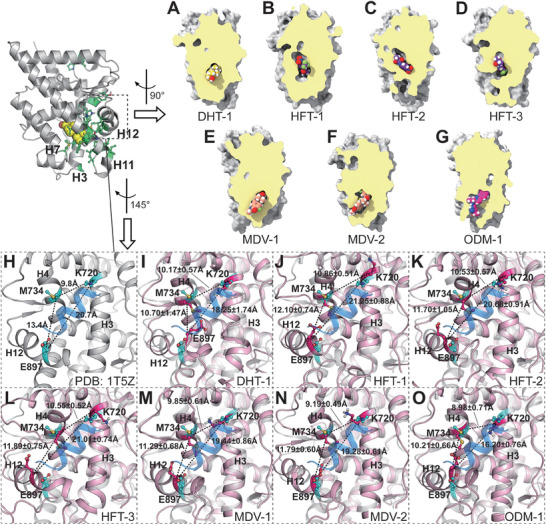
The vertical profile plots illustrating the spatial occupancy of the dominant binding conformations (or docked binding conformations) of various ligands within the AR‐LBD. A) The crystallographic conformation of DHT in complex with the AR‐LBD (PDB: 1T5Z), with the activity‐relevant hydrophobic residues shown as green ball‐stick model. B–D) Three dominant binding conformations of HFT observed in the FM simulations, which are designated as HFT‐1, HFT‐2, and HFT‐3. E,F) Two dominant binding conformations of MDV3100 observed in the FM simulations, labeled as MDV‐1 and MDV‐2. G) The dominant binding conformation (ODM‐1, Minimum 1 in Figure 2D) of ODM201 observed in the FM simulations. H) Positions of three key residues (colored cyan) in the AF2 region in the crystal structure (PDB: 1T5Z); the coactivator is shown as blue cartoon phantoms. I–O) The positions of three key residues (colored hot pink) in the AF2 region of the dominant binding conformations of the four ligands; the crystal structures and coactivators are shown in gray and blue cartoon phantoms, respectively. The average and standard deviation of the interatomic distance of Cα in the conformational ensemble are annotated besides the dash line.

We then aimed to compare the ligand binding differences between the three different antagonists, as they exhibited different sensitivity toward drug‐resistant mutations.^[^ ^8^, ^29^ ^]^ The region highlighted by the contact number in the context of the FM sampling displays a spatial alignment with hydrophobic residues associated with activity (Figure S2A,B, Supporting Information). Herein, eight residues are concurrently present in both residue sets (A698, L704, M780, F876, L880, L881, V889, and F891, as presented in Figure S2C, Supporting Information). This observation implies that those activity‐relevant hydrophobic interactions, within the binding conformations with lower contact number values, are more susceptible to disruption. Thus, this kind of binding conformations are more likely to exert antagonistic functionality. Referring to Figure 2, it becomes evident that the dominant binding conformations of DHT are predominantly distributed within the regions of higher contact values, while the dominant binding conformations of ODM201 are exclusively localized within the regions of very low contact values. This straight‐forward observation supports our assessment. On the other hand, the scenario involving HFT and MDV3100 is quite complex, with their binding conformations distributed across both high and low contact value regions, indicating the presence of both antagonistic and non‐antagonistic components within the binding conformations of these two ligands. In convenience of visualization and comparison, we constructed the longitudinal profile diagrams of the representative complexes to compare the induced‐fit effects on the AR‐LBP, as depicted in **Figure** **5**. Remarkable differences in the binding pocket profiles are observed when different ligands are bound. Upon the DHT binding (Figure 5A), the binding pocket adopts a “closed” configuration, with DHT deeply embedded within the interior of the AR‐LBD. A similar characteristic of the AR‐LBP is observed in HFT‐1 (Figure 5B). Following our proposed hypothesis, typical antagonistic binding conformations are exemplified by HFT‐2, HFT‐3, and ODM‐1 (Figure 5C,D,G), wherein the AR‐LBP adopts an “open” configuration due to the unfolding of helix‐11. In the two dominant binding conformations of MDV3100, the orientation and binding location of the ligand are similar (Figure 5E,F; Figure S3, Supporting Information), with the main difference being the conformation of the unfolded C‐terminus of helix‐11. In MDV‐1, the C‐terminus of helix‐11 is oriented away from the AR‐LBP, whereas in MDV‐2, the C‐terminus of helix‐11 folds inward into the interior of the AR‐LBP, resulting in a higher contact value in this binding conformation (Figure S3, Supporting Information). In terms of the pocket shape, it is hard to define these two conformations as “open” or “closed”, therefore merely based on contact number values, we cannot determine which binding conformation of MDV is the non‐antagonistic one.

Drug‐resistant mutations in the AR‐LBD such as W741C, H874Y, F876L, and T877A can confer AR agonistic properties on HFT and MDV3100, but they hardly affect ODM201. According to our calculation, these resistant mutations might incline HFT and MDV3100 to adopt non‐antagonistic binding conformations with higher contact number values during binding, leading to a shift in the distribution of conformations. In the case of ODM201, its binding conformations are exclusively distributed within the regions of lower contact values. Therefore, even if resistant mutations cause a shift in the distribution of its binding conformations, the shift may be not enough to convert the binding conformation of ODM201 into functionally non‐antagonistic. However, the hypothesis appears to invert the cause and effect relationship, necessitating further exploration.

The transcriptional activation effect of AR is directly influenced by the binding of coactivators, hence the relative positional relationship among the three anchor residues (i.e., K720, M734, and E897) is intimately linked to AR activity. To investigate whether the aforementioned conformations are antagonistic, we computed the average distance between the Cα atoms of these three residues across the conformational ensembles represented by the aforementioned conformations. We used the crystal structure as a reference for comparison, as depicted in Figure 5H–O. Notably, in the ODM‐1 conformation ensemble, the Cα interatomic distance between K720 and E897 is significantly shorter than that observed in the crystal structure and other conformational ensembles. This indicates that the AF2 region is insufficiently accommodated by the coactivator (Figure 5O), thus confirming ODM‐1 as an antagonistic conformation. In contrast, for the dominant binding conformations of DHT, HFT, and MDV3100 (Figure 5I–N), the Cα interatomic distances of the three residues do not differ significantly from those in the crystal structure (Figure 5H). However, it is worth noting that the orientation of H12 in the HFT‐3 conformational ensemble differs significantly from that in the crystal structure, and the Cα atom of E897 exhibits a tendency to move away from the coactivator compared to the crystal structure (Figure 5L). suggesting that this conformation may also be antagonistic. Unfortunately, the two dominant binding conformations of MDV3100 do not exhibit particularly noticeable differences in the AF2 region compared to the crystal structure (Figure 5M,N), thus making it challenging to determine which conformation is antagonistic based on the current findings.

### 2.5 Discovery of Novel Anti‐Resistant AR Antagonists

**Figure 6:**
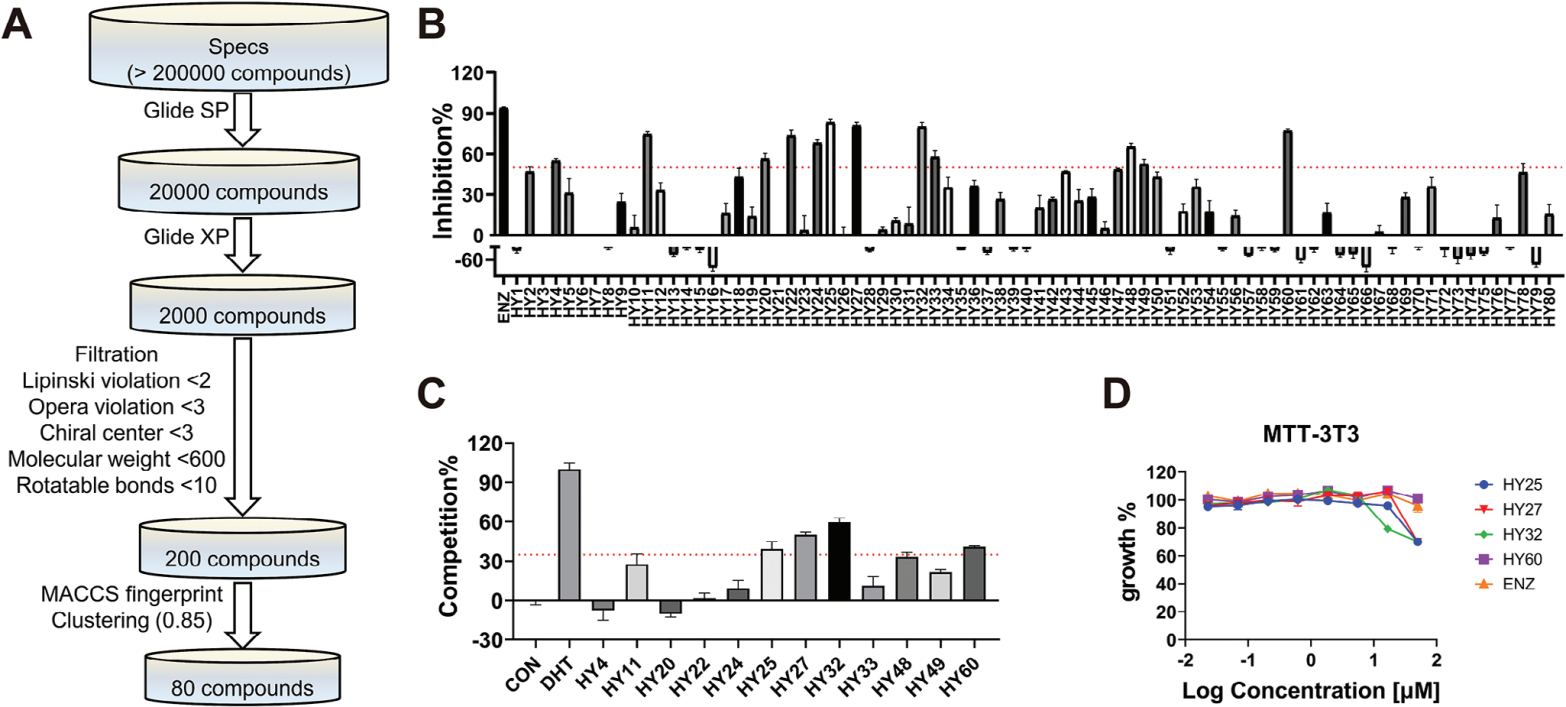
The identification of three novel anti‐resistance antagonists. A) The virtual screening workflow based on the complex structure ODM‐1. B) Transcriptional antagonistic activity of the 80 compounds purchased based on the virtual screening results against AR at 10 µm concentration (*n* = 3). C) Binding activity of 12 potentially active compounds to the AR‐LBD at 10 µm concentration (*n* = 3). D) Anti‐proliferation effects of HY25, HY27, HY32, and HY60 on mouse fibroblast NIH3T3 cell.

To validate that the anti‐resistant properties of ODM201 are closely linked to its distinctive binding mode, the complex structure of ODM‐1 was used to generate the molecular docking grid for VS aiming to identify entirely novel AR antagonists with similar anti‐resistant properties. The workflow for VS is outlined in **Figure** **6**A. At first, the 80 compounds obtained from the VS were subjected to a reporter gene assay to evaluate their antagonistic activity against AR. As depicted in Figure 6B, compared to the control group of enzalutamide, 12 compounds at 10 µm exhibited significant AR transcriptional antagonism activity (inhibition% > 50%). These compounds then underwent in vitro competition experiments at the concentration of 10 µM to assess their binding affinity to the AR‐LBP. The results indicated that out of these 12 compounds, four compounds, HY25, HY27, HY32, and HY60, demonstrated specific AR binding activity (competition% > 35%, as shown in Figure 6C). To rule out the inherent toxicity of the tested compounds, the impact of these four compounds on the proliferation of mouse fibroblasts cell line NIH3T3 was assessed. As illustrated in Figure 6D, these four compounds showed minor effect on the growth of NIH3T3, exhibiting relatively safe profiles. In general, the preliminary bioassays identified four safe and specific AR antagonists.

**Figure 7:**
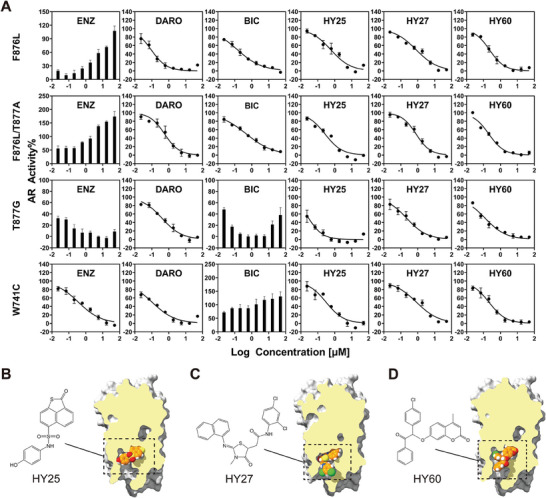
The antagonistic activity against typical AR mutants and the predicted binding conformation of three novel anti‐resistance antagonists. A) Transcriptional activity of MDV3100 (ENZ), ODM201 (DARO), bicalutamide (BIC), HY25, HY27, and HY60 on the ARF876L, ARF876L/T877A, ART877G, and ARW741C mutants (*n* = 3). All results were presented as SEM ± means. B–D) The 2D structures and docked binding conformations of the three lead compounds obtained from the virtual screening. In the Glide docking procedure, only the ligand's flexibility was considered.

Various AR mutants have been reported or clinically detected in patients following the treatment with AR antagonists. It has been reported that the mutation F876L confers resistance to the second‐generation antiandrogens enzalutamide and ARN‐509 and has been detected in the plasma DNA of CRPC patients previously treated with enzalutamide and apalutamide.^[^ ^31^ ^]^ Another common AR mutation T877A is associated with the development of resistance to the first‐generation antiandrogen hydroxyflutamide and promotes prostate cancer cell growth.^[^ ^32^ ^]^ Enzalutamide and apalutamide retain partial antagonistic activity against the mutation of F876L, while the double mutation F876L/T877A further reduces their antagonistic activity.^[^ ^33^ ^]^ Additionally, a previously uncharacterized variant, T877G, is predicted to be partially activated by enzalutamide, and the W741C/L mutation causes bicalutamide to act as an agonist.^[^ ^34^ ^]^ To test whether the above mutations exhibit cross‐resistance to compounds HY25, HY27, HY32, and HY60, a dual luciferase reporter gene assay was conducted in AR‐negative PC3 cells to investigate the transcriptional activity of four AR mutants AR^F876L^, AR^F876L/T877A^, AR^W741C^, and AR^T877G^ in response to MDV3100, ODM201, bicalutamide, and the four newly discovered AR antagonists. As shown in **Figure** **7**A, MDV3100 exhibited significant transcriptional agonist activity toward AR^F876L^ and AR^F876L/T877A^, and partial agonistic activity against AR^T877G^. The first‐generation AR antagonist bicalutamide exhibited agonistic effect on the AR^W741C^ mutant and behaved as a partial agonist on the AR^T877G^ mutant. ODM201 displayed highly effective transcriptional antagonism against all tested variants. Strikingly, except for HY32, three of the lead compounds (i.e., HY25, HY27, and HY60) exhibited explicit transcriptional agonistic activity toward all the AR mutants, too (Figure 7A). As indicated in **Table** **2**, HY25 exhibited IC_50_ values of 0.35, 0.35, 0.06, and 0.33 µm against AR^F876L^, AR^F876L/T877A^, AR^T877G^, and AR^W741C^, respectively. Compound HY27 showed IC_50_ values of 0.65, 0.71, 0.30, and 0.75 µm, and compound HY60 showed IC_50_ values of 0.16, 0.18, 0.12, and 0.22 µm. Furthermore, HY25, HY27, and HY60 bound to the AR‐LBP in a dose‐dependent manner (Table 2, IC_50 HY25_ = 14.96 µm, IC_50 HY27_ = 3.73 µm, and IC_50 HY60_ = 5.96 µm), indicating these three lead compounds directly target the AR‐LBP. According to the binding conformations generated by molecular docking, these three compounds are well accommodated within the ODM201‐induced binding pocket (Figure 7B–D).

**Table 2:** The results of transcriptional activity toward the wild‐type AR and resistant mutants for the hit compounds.

Ligands	AR binding IC_50_[µm]	AR transcriptional activity IC_50_ [µm]				
		WT‐AR	F876L	F876L/T877A	T877G	W741C
HY25	14.96 ± 1.73	1.17 ± 0.33	0.35 ± 0.13	0.35 ± 0.04	0.06 ± 0.06	0.33 ± 0.08
HY27	3.73 ± 0.85	1.87 ± 0.74	0.65 ± 0.14	0.71 ± 0.08	0.30 ± 0.22	0.75 ± 0.14
HY32	4.60 ± 1.12	0.14 ± 0.03	NA^b)^	NA^b)^	NA^b)^	NA^b)^
HY60	5.96 ± 2.51	0.76 ± 0.35	0.16 ± 0.04	0.18 ± 0.01	0.12 ± 0.04	0.22 ± 0.04
MDV	3.68 ± 0.71	0.15 ± 0.06	Agonist	Agonist	Partial agonist	0.29 ± 0.11
ODM	ND^a)^	0.11 ± 0.05	0.11 ± 0.06	0.37 ± 0.15	0.32 ± 0.08	0.16 ± 0.08
BIC	ND^a)^	ND^a)^	0.14 ± 0.01	0.31 ± 0.16	Partial agonist	Agonist

^a)^ ND: Not determined;

^b)^ NA: No activity.

## 3 Conclusion

In this study, we utilized the powerful sampling method known as funnel metadynamics to rigorously elucidate the pathways and binding free energies of the agonist DHT and three clinically approved antagonists (HFT, MDV3100, and ODM201) as they enter/exit the AR‐LBP. Thorough the analysis of the binding free energy surfaces and corresponding conformational ensembles generated by the FM sampling, we aggregated the preferred binding conformations for each ligand. Our analysis revealed that the differential regulation of residue‐residue interactions within the AR‐LBP by the ligands was concentrated around several hydrophobic residues, particularly those centered on the C‐terminus of helix‐11. Further conventional simulations and point‐mutation bioassays highlighted the significance of these hydrophobic residue interactions in maintaining AR activity. We also observed strengthened couplings between the AR‐LBP, AF2, and BF3 upon the DHT binding, and by contrast, these couplings were abolished upon the binding of antagonists. Comparative analysis of vertical profiles illustrated a dramatic induced‐fit effect of the bound ligand on the shape of the AR‐LBP. Noticeable variations in the pocket shape of the AR‐LBP were observed between agonists and antagonists, as well as among different binding modes of the same ligand. Unlike HFT and MDV3100, the pocket was observed only in the “open” state upon the binding of ODM201, possibly contributing to its insensitivity to drug resistant mutations. Based on our theoretical findings, we conducted VS on the basis of the dominant binding conformation of ODM201, and three lead compounds (HY25, HY27, and HY60) were identified that exhibited favorable binding affinity and antagonistic activity toward the AR‐LBP. Furthermore, these three compounds effectively counteract representative AR mutations (specifically, in this study, F876L, F876L/T877A, T877G, and W741C) arising from prolonged clinical use of first and second‐generation AR antagonists.

The mechanisms discussed in this study totally differ from previous focus in nuclear receptor system research, which primarily centered on the polar interactions between ligands and binding pocket residues. Instead, within the scope of this research, we found that the induced ‐fit effects on the AR‐LBP, as well as the hydrophobic residue interactions, were sufficient to determine AR transcriptional activity. Therefore, specific ligand‐receptor polar interactions were not addressed in this study. As a result, regarding the two questions mentioned in the introduction chapter, the first one “how antagonists regulate the conformation of the AR‐LBD to render it inactive” was well answered by our simulation and experimental results. Although the second question “how mutations in specific residues result in the conversion of certain antagonists into agonists” still needs further investigation, the unique binding mode of ODM201 identified by the FM simulation provides an applicable structural basis for searching novel anti‐resistant antagonists. Along this line, even a straight‐forward VS protocol achieved a satisfactory hit rate. Furthermore, the strategies and approaches employed in this study offer valuable insights for the discovery and development of antagonists targeting other nuclear receptors.

## 4 Experimental Section

### System Preparation

The complex structure of the agonist DHT with the AR‐LBD was obtained from the RCSB database (PDB: 1T5Z). The complex of the antagonist HFT with the T877A mutant of the AR‐LBD was previously reported in RCSB (PDB: 2AX6). The structure was downloaded, and the mutated residues were edited to their wild‐type counterparts using Schrodinger 2020. The molecular docking grid for the ligand‐binding pocket was generated using this edited structure. The antagonists MDV3100 and ODM201 were docked into the LBP using the *Glide* program in Schrodinger, resulting in the generation of two complex structures of the antagonists bound to the AR‐LBD. For each system, co‐activators and other irrelevant parts were removed from the crystal structure. The missing heavy atoms were added, and any unreasonable regions in the structure were optimized using the *Protein Preparation Wizard* in Schrodinger.

The partial atom charges of the ligands were calculated using the restrained electrostatic potential (RESP) protocol.^[^ ^35^ ^]^ The electrostatic potentials were computed at the HF/6‐31G level using Gaussian16. The force field parameters for the protein and ligands were generated using the *Antechamber* module in AMBER18,^[^ ^36^ ^]^ specifically the FF14SB force field^[^ ^37^ ^]^ for the protein and the general Amber force field (GAFF)^[^ ^38^ ^]^ for the ligands. The system was solvated using the *tleap* module, and an explicit solvent model (TIP3P) was used for water molecules. The solvent box was set to have a boundary distance of 12 Å from the protein. Additionally, counter ions (Na^+^ and Cl^−^) were added to balance the charge of the system.

### The Random Accelerated Molecular Dynamics Simulation

Each system was minimized using the *pmemd* program from the AMBER18^[^ ^36^ ^]^ software package. Initially, a restraint force of 10.0 kcal mol^‒1^ Å^‒1^ was applied to the protein and ligands. The system underwent 5000 cycles of steepest descent minimization followed by 5000 cycles of conjugate gradient minimization to optimize the solvent and ions. Subsequently, all restraint forces were removed, and the system underwent an additional 5000 cycles of steepest descent minimization and 5000 cycles of conjugate gradient minimization. Next, the heating, equilibration, and RAMD sampling were executed by NAMD2.12 package.^[^ ^39^ ^]^ Each minimized system was heated from 0 to 300 K over a period of 20 ps and then equilibrated for 10 ns with the Langevin thermostat and Nose‐Hoover Langevin pressure control in the NPT (P = 1 atm and T = 300 K) ensemble.

In the RAMD simulation,^[^ ^26^ ^]^ an artificial force (16 kcal mol^‒1^ Å^‒1^) with a random direction was applied to the center of mass of the ligand. At every checkpoint (per 100 fs), if the distance traveled by the center of mass of the ligand exceeds the threshold (0.025 Å), the direction of the artificial force remains unchanged in the next period. Otherwise, the direction of the artificial force was randomly changed in the next period. For each system, 40 parallel replicas of RAMD simulations were produced. The trajectories were visualized using VMD for examining the dissociation pathways of the ligands. Finally, the dissociation pathways of each ligand were summarized.

### Funnel Metadynamics Simulation

To accurately calculate the free energy profile of each system, the GROMACS 2018 package^[^ ^40^ ^]^ and PLUMED2.5 plugin^[^ ^41^ ^]^ were utilized to perform funnel metadynamics.^[^ ^22^ ^]^ The restraint region (Figure S1, Supporting Information) was set according to the RAMD result which pointed out that the channel surrounded by helix‐3, helix‐7, and helix‐11 should be the dominant pathway for ligand entrance/exit. Three parameters Zcc, Rcyl, and α, which defined the shape of the funnel restraint, were set as 0.5 nm, 0.1 nm and 1.1 rad, respectively. Two CVs were defined to better describe the interaction between the ligand and AR‐LBD. CV1 was defined as the distance between the center‐of‐mass of the ligand and the protein. CV2 was defined as the compactness between the channel residues, and the atom‐pairs which were in native contact within 4.5 Å in the crystal structure (PDB: 1T5Z) were chosen to calculate the contact value,^[^ ^42^ ^]^ including atom pairs on the following residues: F697‐L700, F697‐L701, F697‐K777, F697‐S778, F697‐R779, F697‐V887, A698‐V887, A698‐V889, L700‐S703, L700‐704, L700‐S778, L701‐S778, L701‐M780, L701‐F876, L701‐L880, L701‐V889, L701‐D890, L704‐M780, S778‐F876, R779‐F876, M780‐F876, F876‐D879, F876‐L880, L880‐K883, L880‐S884, L880‐V887, L880‐V889, L880‐F891, L881‐F891, K883‐M886, K883‐V887, S884‐S888, S884‐V889, and S884‐F891. The contact value S(X) of a certain conformation X was calculate as following equation:

(1) $$S\;\left(X\right)=\sum_{i=1}^{m}\frac{1}{1+e^{\beta \left(r_{i}-\lambda r_{0}\right)}}$$

where *β* and *λ* were set as 50.0 nm^−1^ and 1.8, respectively. The value *r*_i_ is the atom distance of *i*th atom‐pair, *r*_0_ is the reference value which was set as 0.45 nm, *m* is the total number of native contact atom‐pairs, and herein totally 254 native pairs were identified by the in‐house scripts.

The Gaussian width of CV1 and CV2 were set to 0.1 and 10, respectively. The Gaussian height was set to 1.0 kJ mol^−1^. The period of adding bias was set as 2 ps. The bias factor, a parameter used to decrease the added bias in a history dependent manner, was set to 16 to improve the convergence. Each system was sampled for over 1000 ns, and the added biases were deducted to recover the original free energy surface. The convergence of the sampling was tested based on the free energy landscape variation as a function of simulation time (Figure S4, Supporting Information).

### Conventional MD Simulation

In order to prove the relationship between hydrophobic contacts and AR activity, conventional MD trajectories were launched by AMBER18 package^[^ ^36^ ^]^ for the WT‐AR and several designed mutants. Each production run were carried out for 1 µs, and the frames in last 200 ns were taken for analysis. For each system, two variables were calculated, including the root mean square deviation (RMSD) of the AF2 region (main‐chain heavy atoms) and the increment of root mean square fluctuation (ΔRMSF). The latter was defined as following:

(2) $$\Delta \mathrm{RMSF}=\frac{RMSF_{mut}-RMSF_{wt}}{N}$$

where *RMSF*_mut_ and *RMSF*_wt_ are the summation of RMSF value of the AF2 main‐chain heavy atoms, *N* is the total number of the AF2 main‐chain heavy atoms.

### Virtual Screening

The representative binding conformation of ODM201 (denoted as ODM‐1) was used to generate the docking grid using the *Receptor Grid Generation* module in Schrodinger. The *Glide* docking program was then utilized to screen the Specs database. Initially, over 200 000 compounds were prepared using the *LigPrep* module in Schrodinger by generating possible protonation states at pH = 7.0 ± 2.0. The prepared ligand dataset was then screened by using *Glide* with the SP scoring, and the top 20000 scored compounds were subjected to a XP precision re‐docking procedure using Glide, followed by scoring‐based ranking. Subsequently, the top 2000 compounds were retained and further filtrated by conventional drug‐like properties (Figure 6A), and then the top 200 compounds were clustered based on their MACCS fingerprint (similarity threshold = 0.85). Within each cluster, the compound with the best docking score was retained. Ultimately, the 80 compounds with the most favorable docking scores in the final list were were purchased for bioassays.

### Materials for Bioassays

Plasmid pGL4‐ARR2PB‐luc was constructed by cloning ARR2PB fragment into pGL4.18 [luc2P/Neo] (#E6731, Promega). Plasmid pCMV‐hAR (#89 078, Addgene) was a gift from Elizabeth Wilson. Plasmids hAR^F876L^, hAR^F876L/T877A^, hAR^T877G^, and hAR^W741C^ were constructed by site‐directed mutagenesis of pCMV‐hAR through overlap extension PCR.

PC3 cells were propagated in RPMI‐1640 (#MA0215, Meilunbio) supplemented with 10% fetal bovine serum (FBS, #F8318, Sigma‐Aldrich). NIH3T3 cells were cultured in DMEM (#MA0212, Meilunbio) with 10% FBS. All growth media were supplemented with 1% Penicillin–Streptomycin–Glutamine (#10 378 016, Gibco). Cell cultures were maintained in culture flasks in 5% CO_2_ atmosphere at 37 °C. To avoid the disturbance of relevant endogenous factors in FBS, FBS were stripped with dextran‐coated charcoal (DCC) to remove most of its hormone, growth factors, and cytokines. Phenol red‐free media along with 5% DCC‐stripped serum‐starvation (CSS) were used for treatment studies.

### Cell Proliferation Assays

For cytotoxicity assay, NIH3T3 cells were used to preliminarily rule out the inherent toxicity of the tested compounds. NIH3T3 cells were cultured in DMEM media at a density of 4 × 10^3^ cells per well. After incubation at 37 °C for 24 h, cells were treated with gradient concentrations of indicated compounds for 72 h. 10 µL of 5 mg mL^−1^ MTT solution were added into each well and incubated for 4 h, then 100 µL of triplex solution (10% SDS, 5% isobutyl alcohol, and 0.012 mol L^−1^ HCl) were added to dissolve the formazan crystals, and the plates were then further retained in an incubator overnight. The absorbance at 570 nm was measured with the reference wavelength at 650 nm using a Synergy H1 microplate reader (BioTek).

### AR Competitor Assay

The competitive binding of the tested compounds toward AR was assessed with the PolarScreen Androgen Receptor Competitor Assay Kit, Green (#A15880, Thermo Scientific) as the instruction of the manufacturer. The assay was used to investigate the competitive binding of the test compound to the LBP site beforehand occupied by a high‐affinity fluorophore ligand. Briefly, the test compound solutions in DMSO were diluted in AR green buffer to achieve 2× test concentrations and placed in a 384‐well plate with 10 µL volume capacity, followed by adding a 2× AR‐LBD/ Fluormone mix. Plates were then incubated protected from light for at least 4 h. The FP value (mP) of each well was measured with a Synergy H1 microplate reader using an optic module with excitation at 485 and emission at 535 nm (BioTek).

### AR Transcriptional Activity Assay

PC3 cells were starved in RPMI media with 5% DCC‐stripped serum for 3 days before seeding. The PC3 cells (1 × 10^4^ cells per well, 96 well) were then transfected with hAR plasmid (50 ng), pGL4‐ARR2PB‐Luc (20 ng), and 5 ng Renilla. Cells were treated with the tested compounds in the absence or presence of 1 nm DHT inducing a submaximal reporter gene activation in steroid‐free assay medium and incubated for 24 h. Luciferase activity was measured using a Dual‐Lumi II Luciferase Assay Kit (#E1960, Promega) as per the manufacture. Luminescence was measured with Synergy H1 (BioTek, USA).

### Study on AR Mutants

PC3 cells were seeded and transfected as described above. The PC3 cells were then transfected with plasmids expressing hAR^F876L^, AR^F876L/T877A^, AR^T877G^, or AR^W741C^ mutant (50 ng), pGL4‐ARR2PB‐Luc (20 ng) and Renilla (5 ng). Cells were treated with gradient concentrations of the test compounds in the absence or presence of 0.5 nm DHT inducing a submaximal reporter gene activation in steroid‐free assay medium and incubated for 24 h. Luciferase activity was measured as described above.

### Statistical Analysis

All the experiments were repeated at least three times independently. Data were analyzed, and dose‐response curves were generated using GraphPad Prism 8 software (GraphPad Software, La Jolla, CA, USA), and results were presented as mean ± SEM.

## Conflict of Interest

The authors declare no conflict of interest.

## Supporting information

Supporting Information

## Data Availability

The data that support the findings of this study are available from the corresponding author upon reasonable request.
