# Supplementary material for: Discovery of Novel Anti‐Resistance AR Antagonists Guided by Funnel Metadynamics Simulation
Source: Adv Sci (Weinh). 2024 Mar 13;11(19):2309261. doi: 10.1002/advs.202309261 (PMC11109662; doi:10.1002/advs.202309261)
Supplement: Supplementary file 1 — Supporting Information [file ADVS-11-2309261-s001.pdf]

ADVANCED SCIENCE
Open Access

Supporting Information

for *Adv. Sci.*, DOI 10.1002/advs.202309261

Discovery of Novel Anti-Resistance AR Antagonists Guided by Funnel Metadynamics Simulation

Haiyi Chen, Yuxin Zhou, Xinyue Wang, Xin Chai, Zhe Wang, Ercheng Wang, Lei Xu, Tingjun Hou, Dan Li* and Mojie Duan**

Supporting Information

Table S1. The dissociation pathways of the four ligands in RAMD simulations were recorded across all trajectories[a].

	Ligands			
Index	DHT	HFT	MDV3100	ODM201
1	I	II	II	I
2	I	II	II	II
3	IV	II	II	I
4	III	I	II	II
5	I	II	II	II
6	IV	I	II	II
7	II	I	II	III
8	II	II	II	II
9	I	I	II	IV
10	V	II	II	II
11	II	II	II	I
12	II	I	II	I
13	II	II	II	II
14	II	II	II	II
15	VI	II	II	II
16	II	II	II	I
17	II	II	II	II
18	II	III	II	II
19	II	I	II	II
20	II	II	II	II
21	II	III	II	II
22	I	I	II	II
23	II	III	II	II
24	II	II	II	I
25	II	II	II	II
26	II	II	II	III
27	II	I	II	II
28	II	II	II	II
29	I	I	II	II
30	II	II	II	I
31	II	I	II	II
32	II	IV	II	II
33	II	II	II	IV
34	II	II	II	II
35	II	II	II	II
36	I	II	II	II
37	II	II	II	II

38	II	II	II	II
39	II	II	II	II
40	II	II	II	II

[a]Four dissociation pathways were defined as follows: I) the loop region between helix-11 and helix-12; II) the channel formed by helix-3, helix-7, and helix-11, which is also the most common ligand entrance and exit pathway in the nuclear receptor LBD domain; III) the gap between helix-1 and helix-3; IV) the gap between helix-3 and helix-12. Trajectories dissociating along pathway II, as indicated in the table, are highlighted.

Table S2. The calculation results of conventional MD trajectories. The calculation protocols of RMSD and ΔRMSF are given in Materials and Methods chapter.

	RMSD	ΔRMSF
WT	2.05±0.08	-
M780A_F876A	2.74±0.15	0.11
W739A_L907A	2.42±0.09	0.06
F876A_L907A	2.57±0.07	0.03
L881A_M895A	2.50±0.10	0.13
L707A_W741A	2.57±0.08	0.14
M745A_L873A	2.76±0.09	0.16
H874A_F876A	2.90±0.10	0.07
M895A_I899A	2.80±0.13	0.01

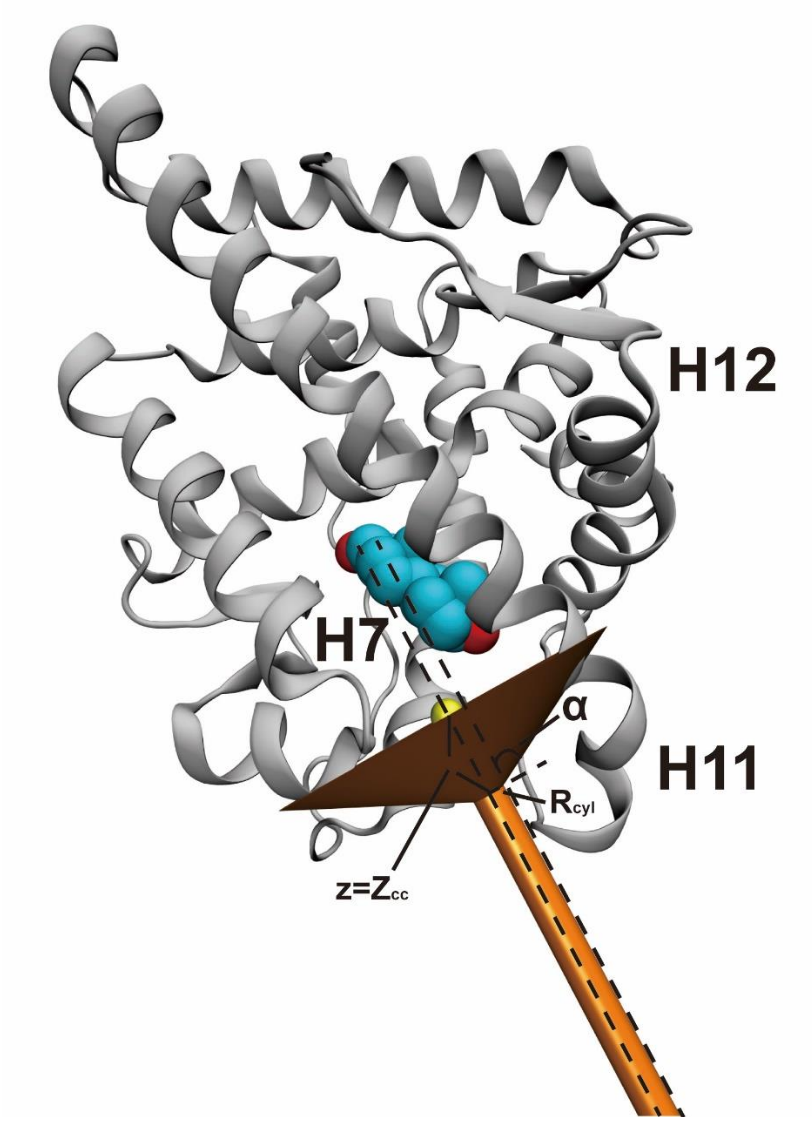

Figure S1. The Funnel restraint setting of the system. The detailed values of R_{cyl}, Z_{cc} and α are given in Methods section.

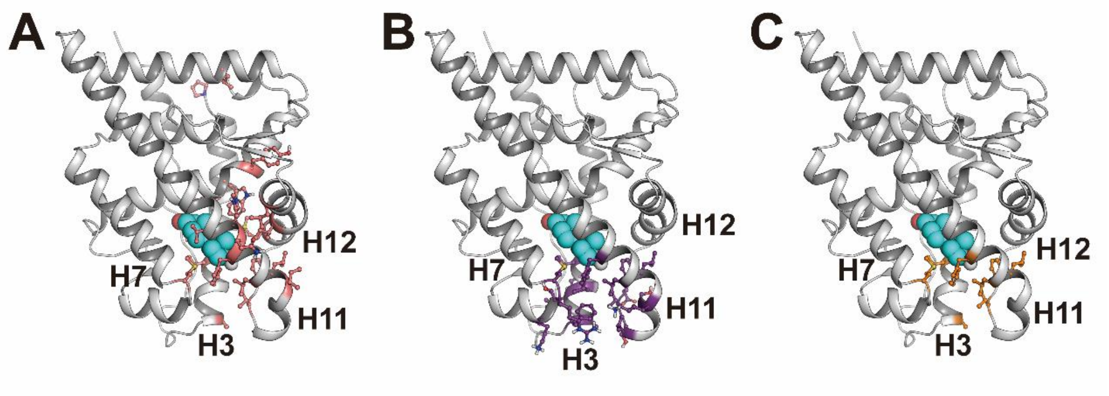

Figure S2. The distribution of crucial residues involved in this study on the AR-LBD. The complex utilized in this figure is the crystal structures of DHT and AR-LBD (PDB: 1T5Z), with the protein represented in white cartoon form and ligand carbon and oxygen atoms shown as blue and red spheres, respectively. **(A)** The hydrophobic residues that show a noticeable reduction in interactions upon antagonist binding, as mentioned in **Fig. 3B**, are highlighted in the pink region. **(B)** The residues involved in the contact value calculation of CV2 in the FM sampling are illustrated in the purple region. **(C)** The overlapping segment of residues from the aforementioned two sets is indicated in the orange region.

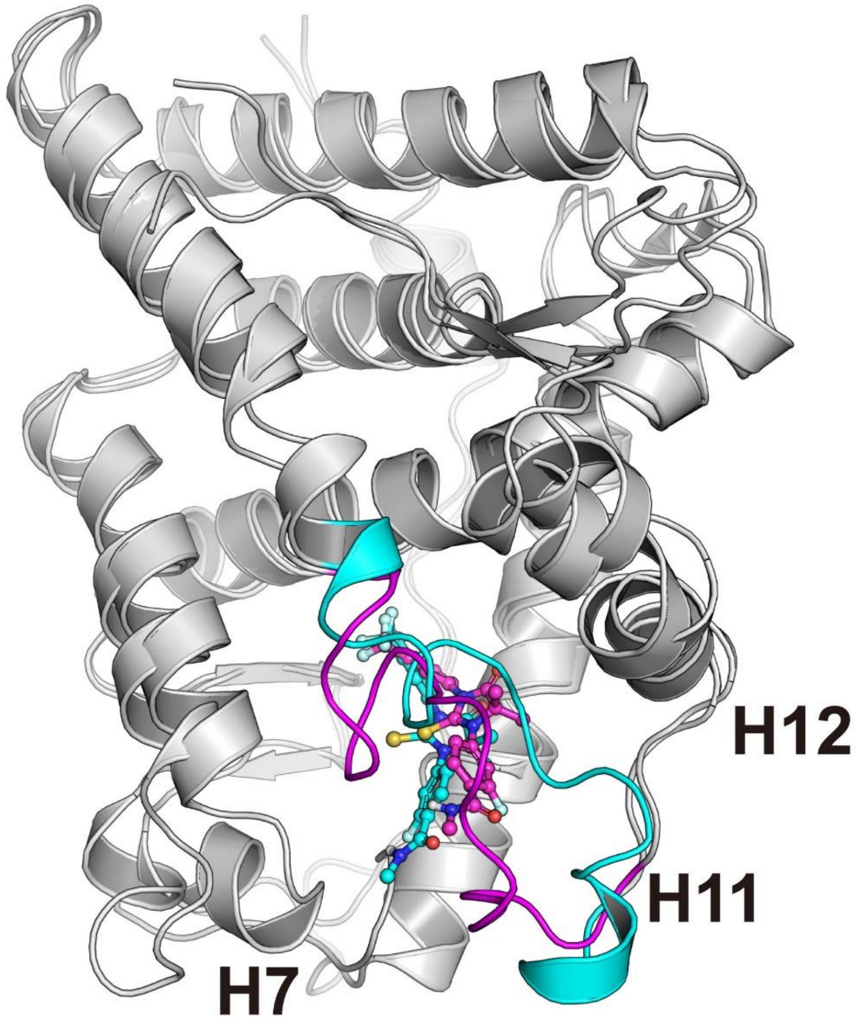

Figure S3. The alignment of binding conformations MDV-1 and MDV-2. The ligands are given as ball-stick model. The ligand and the C-terminus of helix-11 (residue 870~889) are colored in magenta/cyan in MDV-1/MDV-2, respectively.

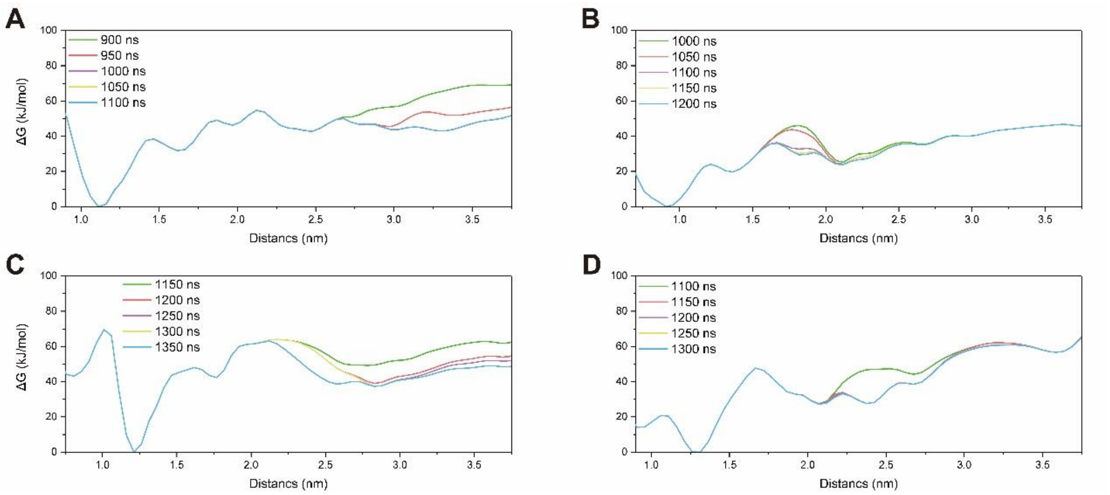

Figure S4. The FM sampling convergence was reached for each system. (**A**) DHT and AR-LBD, (**B**) HFT and AR-LBD, (**C**) MDV3100 and AR-LBD, (**D**) ODM201 and AR-LBD.
